# Muscone Promotes PINK1/Parkin-Associated Mitophagy to Suppress NLRP3 Inflammasome Activation: Implications for Endotoxemia Therapy

**DOI:** 10.3390/ph19060816

**Published:** 2026-05-23

**Authors:** Ziwei Yan, Minrui Li, Dan Li, Wentian Hua, Haoxue Cao, Yufei Li, Li Che, Xiyi Chen, Zhicheng Lai, Yi Wang, Guofang Shen, Jing Qian

**Affiliations:** 1 Pharmaceutical Informatics Institute, School of Pharmacy, Zhejiang University, Hangzhou 310058, China; yanziwei@zju.edu.cn (Z.Y.); zjuwangyi@zju.edu.cn (Y.W.); 2 Xiamen Traditional Chinese Medicine Co., Ltd., Xiamen 361100, China; 3 Innovation Institute for Artificial Intelligence in Medicine, Zhejiang University, Hangzhou 310018, China; 4 Jinan Microecological Biomedicine Shandong Laboratory, Jinan 250118, China; 5 Hangzhou Institute for Food and Drug Control, Hangzhou 310022, China

**Keywords:** muscone, NLRP3 inflammasome, PINK1/Parkin-associated mitophagy, mitochondrial quality control, macrophages, endotoxemia

## Abstract

**Background**: The NLRP3 inflammasome drives pathological inflammation in various diseases. PINK1/Parkin-associated mitophagy serves as a critical negative regulator of NLRP3 activation, yet pharmacological enhancers remain scarce. Muscone, a natural macrocyclic ketone with blood–brain barrier permeability, exhibits potent anti-inflammatory properties; however, its mechanistic role within the NLRP3-mitophagy axis remains undefined. **Methods**: LPS/ATP-stimulated macrophages were employed to assess stage-specific effects of muscone on NLRP3 priming (NF-κB signaling, NLRP3, and pro-IL-1β expression) and activation (ASC oligomerization, ASC–pro-caspase 1 complex formation, and IL-1β secretion). RNA sequencing and bioinformatic analysis were performed for pathway enrichment. Mitophagy was characterized by MitoSOX Red staining for mt-ROS detection, electron microscopy, Western blotting of LC3B-II in isolated mitochondria and PINK1 and Parkin in whole-cell lysates, and live-cell mitochondria–lysosome tracking. In vivo protective efficacy was assessed in an LPS-induced endotoxemia mouse model. **Results**: Muscone dose-dependently suppressed both the priming and activation stages of the NLRP3 inflammasome, maximally reducing IL-1β secretion by ~60% at 50 μM. Mechanistically, muscone amplified PINK1/Parkin-associated mitophagy, scavenging excessive mt-ROS and attenuating NLRP3 activation. These effects were corroborated by RNA-seq and comprehensive functional assays. In vivo, muscone (30 mg/kg) significantly improved survival (3/8 mice alive at 98 h when all LPS controls had died; 2/8 survived to the 132-h endpoint), with concomitant enhancement of mitophagy markers in peritoneal macrophages. **Conclusions**: Muscone functions as a PINK1/Parkin-associated mitophagy enhancer that maintains mitochondrial quality control during NLRP3-driven inflammatory responses. Its unique macrocyclic structure and blood–brain barrier permeability provide a promising scaffold for developing therapeutics against inflammatory disorders associated with NLRP3 inflammasome activation.

## 1. Introduction

The NOD-like receptor protein 3 (NLRP3) inflammasome is a cytosolic multiprotein complex that serves as a central driver of innate immunity and inflammation [1,2]. Upon activation by pathogen- or damage-associated molecular patterns, it orchestrates caspase-1-dependent maturation of interleukin-1β (IL-1β) and IL-18, as well as pyroptotic cell death—processes implicated in diverse inflammatory disorders, including endotoxemia and sepsis [3]. This canonical activation involves two steps: an initial priming stage driven by nuclear factor kappa B (NF-κB) signaling and a subsequent activation stage triggered by NLRP3-apoptosis-associated speck-like protein containing a CARD (ASC) complex assembly [4]. While the priming stage is well characterized, the precise triggers for activation remain elusive. Nevertheless, mitochondrial-derived danger signals such as mitochondrial reactive oxygen species (mt-ROS) and mitochondrial DNA (mt-DNA) are indispensable, pointing to impaired mitochondrial quality control as a potential regulatory node [5]. Pharmacological strategies targeting this axis, however, remain to be fully explored.

Autophagy is an intracellular catabolic process that degrades cellular components via double-membrane autophagosomes [6]. Among its selective forms, mitochondrial autophagy—termed mitophagy—specifically eliminates damaged mitochondria to preserve cellular homeostasis [7], with the PTEN-induced putative kinase 1 (PINK1)/Parkin pathway being the best-characterized mechanism. This pathway operates as follows: mitochondrial depolarization stabilizes PINK1, which recruits and activates the E3 ubiquitin ligase Parkin; activated Parkin then ubiquitinates outer mitochondrial membrane proteins, tagging them for autophagy adapter recognition, microtubule-associated protein 1 light chain 3 (LC3)-mediated engulfment, and lysosomal degradation [8,9]. Importantly, this quality control mechanism has emerged as a functional suppressor of NLRP3 inflammasome activation, largely by removing the mt-ROS that fuels its assembly [10]. While genetic studies—PINK1 or Parkin knockout in particular—have firmly established this axis [11], pharmacological enhancers of PINK1/Parkin-mediated mitophagy remain scarce, representing a significant unmet therapeutic need.

Muscone (3-methylcyclopentadecan-1-one) is the principal active component of musk (Shexiang, Moschus), a traditional Chinese medicine derived from the abdominal secretions of male musk deer (genus Moschus) [12,13]. This natural macrocyclic ketone exhibits diverse biological activities, including cerebrovascular protection, anti-inflammatory, and neuroprotective effects [14]. Critically, its high lipophilicity and blood–brain barrier permeability [15] position it as a promising scaffold for central nervous system (CNS)-targeted drug development. Regarding NLRP3 inflammasome regulation, muscone has been reported to attenuate neuroinflammation by regulating mitochondrial fission [16] and to relieve inflammatory pain via NLRP3 inflammasome suppression in microglia [17]. However, whether muscone directly engages the mitochondrial quality control machinery to suppress NLRP3 activation remains unknown.

Building upon our prior identification of muscone as an NLRP3 inhibitor from the traditional formula Baobaodan [18], the present study elucidates its molecular mechanism of action. Using LPS/ATP-stimulated macrophages and an endotoxemia mouse model, we demonstrate that muscone functions as a PINK1/Parkin-associated mitophagy enhancer, thereby attenuating NLRP3-driven inflammation through mitochondrial quality control. These findings establish a pharmacological link between mitochondrial clearance and innate immune regulation, with implications for developing CNS-penetrant NLRP3-targeted therapeutics.

## 2. Results

### 2.1. Muscone Suppresses Both Priming and Activation of the NLRP3 Inflammasome

**Figure 1:**
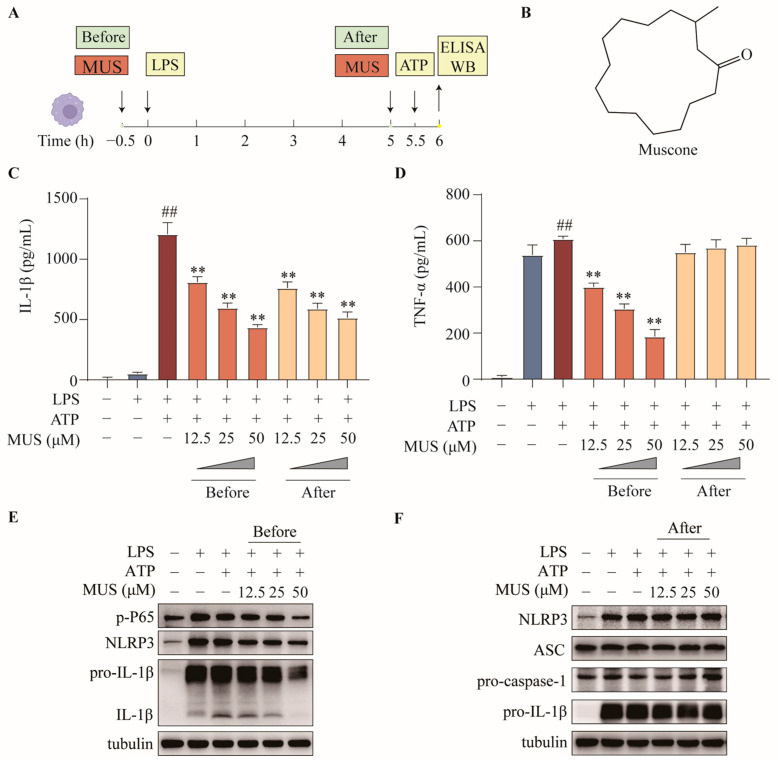
Muscone suppresses NLRP3 inflammasome priming and activation: (**A**) Experimental design: Peritoneal macrophages (PMs) were primed with LPS (500 ng/mL, 5.5 h) followed by ATP (2 mM, 30 min). Muscone (12.5, 25, 50 μM) was added either 0.5 h before LPS (priming stage, “Before”) or 5 h after LPS (activation stage, “After”). (**B**) Chemical structure of muscone. (**C**,**D**) IL-1β (**C**) and TNF-α (**D**) levels in cell culture supernatants measured by ELISA. (**E**) Western blotting of p-P65, NLRP3, pro-IL-1β, and mature IL-1β in cells treated during the priming stage. (**F**) Western blotting of NLRP3, ASC, pro-caspase-1, and pro-IL-1β in cells treated during the activation stage. Representative blots from three independent experiments are shown. Data are presented as mean ± standard error of the mean (SEM). ## *p* ≤ 0.01 vs. control; ** *p* ≤ 0.01 vs. LPS + ATP using one-way ANOVA followed by Tukey’s multiple-comparison post hoc test.

To dissect the stage-specific effects of muscone, we administered it either during priming (“Before”) or after priming (“After”) in LPS/ATP-stimulated macrophages (Figure 1A,B). Since IL-1β production requires full NLRP3 activation, whereas TNF-α reflects NF-κB signaling alone, differential effects on these cytokines indicate distinct mechanisms. ELISA showed that muscone concentration-dependently inhibited IL-1β in both stages (maximal suppression ~60% at 50 μM) and TNF-α during priming (by ~70% at 50 μM), but not during activation, suggesting differential targeting of NF-κB-dependent versus NF-κB-independent mechanisms (Figure 1C,D). These concentrations (12.5–50 μM) were confirmed to be non-cytotoxic to peritoneal macrophages at both 1 h and 6.5 h post-treatment by MTT assay (Figure S1).

Western blotting confirmed these differential effects: muscone added before LPS reduced NLRP3, pro-IL-1β, and mature IL-1β, accompanied by decreased p-P65, indicating NF-κB pathway inhibition (Figure 1E). Conversely, muscone added after LPS priming did not alter NLRP3, pro-IL-1β, or ASC levels (Figure 1F), demonstrating NF-κB-independent suppression of inflammasome activation.

**Figure 2:**
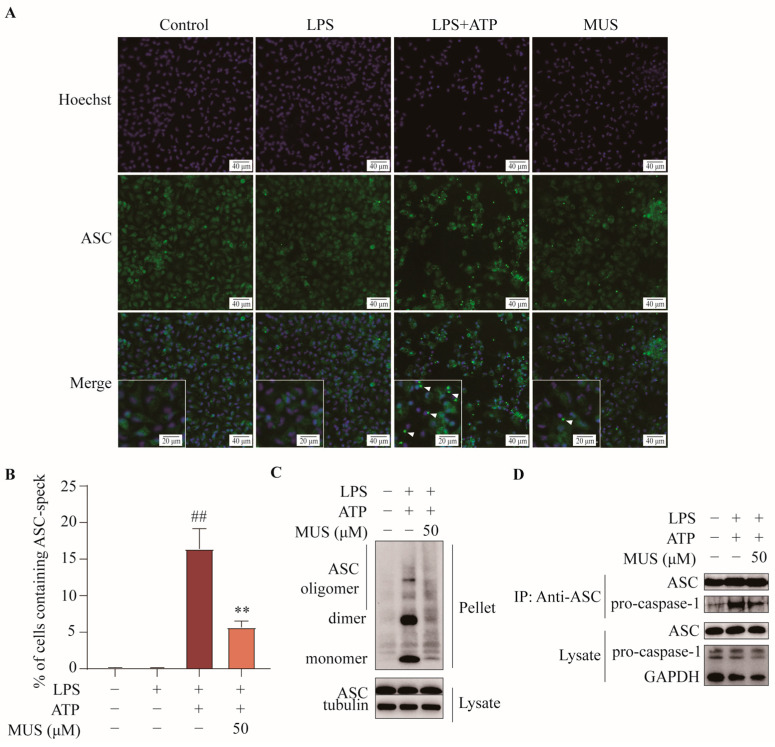
Muscone disrupts NLRP3 inflammasome assembly independently of priming. LPS-primed PMs were treated with muscone (50 μM) for 0.5 h prior to ATP stimulation. (**A**) ASC speck formation assessed by immunofluorescence. White triangles indicate ASC specks. Green: ASC; blue: nuclei (Hoechst). Scale bar: 40 μm (overview), 20 μm (inset). (**B**) Percentage of ASC speck-positive cells quantified using ImageJ version 1.51j8 (*n* = 8 fields per well, 3 biological replicates per group). (**C**) ASC oligomerization detected by Western blotting. (**D**) Interaction between ASC and pro-caspase-1 assessed by co-immunoprecipitation. Representative images from three independent experiments are shown. Data are presented as mean ± SEM. ## *p* ≤ 0.01 vs. Control; ** *p* ≤ 0.01 vs. LPS + ATP using one-way ANOVA followed by Tukey’s multiple-comparison post hoc test.

We next investigated assembly machinery directly by assessing ASC oligomerization, speck formation, and pro-caspase-1 recruitment—hallmark events of NLRP3 activation [19]. Muscone markedly curtailed ASC speck formation (~70% reduction in speck-positive cells, Figure 2A,B) and oligomerization without altering total ASC (Figure 2C). Moreover, muscone disrupted ASC–pro-caspase-1 interaction induced by LPS + ATP (Figure 2D). These findings establish that muscone suppresses NLRP3 activation, yet the underlying mechanism remains elusive.

### 2.2. RNA-Seq Links Muscone’s Suppression of NLRP3 Activation to Mitophagy

**Figure 3:**
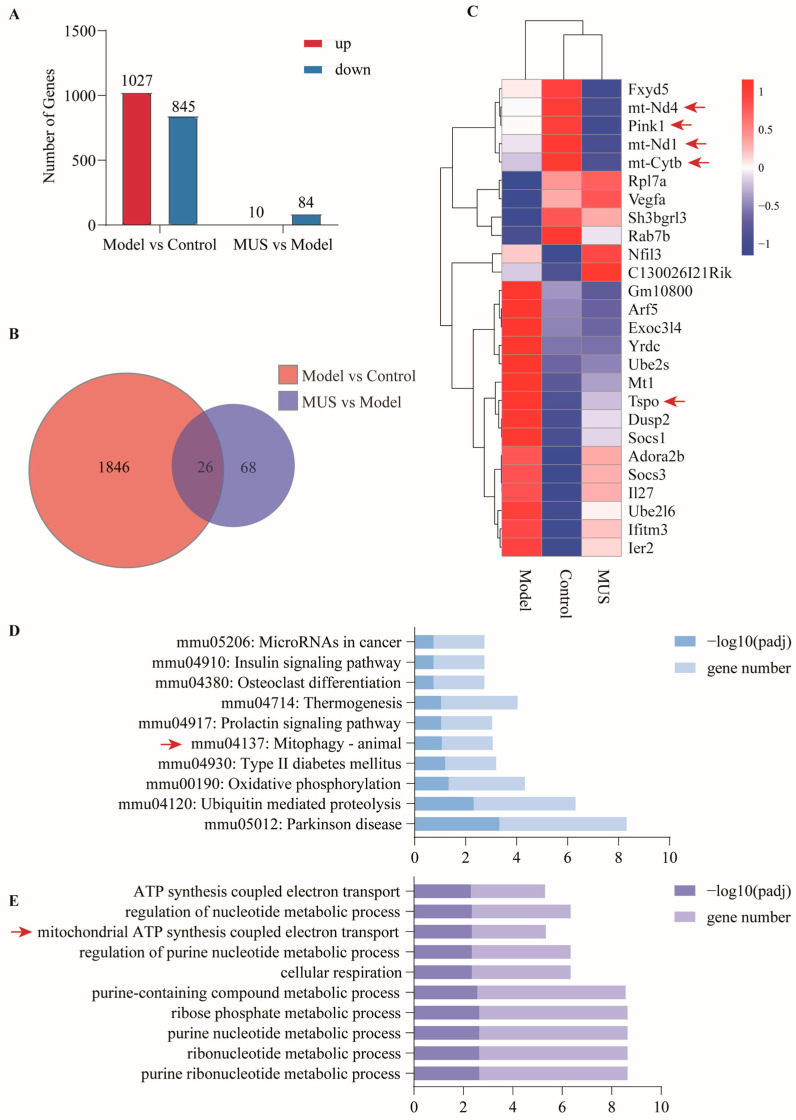
Unbiased RNA-seq identifies mitochondrial quality control as a target of muscone. Untreated (Control), LPS + ATP (Model), and muscone-treated (50 μM, 0.5 h prior to ATP, MUS) cells were harvested for RNA-seq (*n* = 2 per group). (**A**) Number of differentially expressed genes (DEGs) in Model vs. Control and MUS vs. Model comparisons. (**B**) Venn diagram of shared DEGs. (**C**) Hierarchical clustering of shared DEGs; mitochondrial components (mt-Nd4, mt-Nd1, mt-Cytb, and TSPO) and mitophagy regulator PINK1 are indicated. (**D**) Top 10 KEGG pathways; mitophagy highlighted in red arrows. (**E**) Top 10 GO biological process terms; mitochondrial ATP synthesis-coupled electron transport highlighted in red arrows.

To identify potential mediators, RNA-seq was performed on stage 2-restricted conditions on untreated (Control), LPS + ATP (Model), and muscone-treated (50 μM, 0.5 h prior to ATP stimulation, MUS) cells. Comparison of Model vs. Control identified 1872 differentially expressed genes (DEGs; 1027 upregulated and 845 downregulated), whereas MUS vs. Model revealed 94 DEGs (10 upregulated and 84 downregulated) (Figure 3A). Venn analysis identified 26 shared DEGs (Figure 3B), with distinct expression patterns across groups (Figure 3C).

Notably, shared DEGs included genes encoding mitochondrial components (mt-Nd4, mt-Nd1, mt-Cytb, and TSPO) and the mitophagy regulator PINK1 (Figure 3C). KEGG pathway analysis highlighted mitophagy (Figure 3D), while GO analysis identified enrichment in mitochondrial ATP synthesis-coupled electron transport (Figure 3E). These transcriptomic signatures suggest that muscone enhances mitochondrial quality control, which we subsequently validated by functional and imaging assays.

### 2.3. Muscone Attenuates Mitochondrial Reactive Oxygen Species During NLRP3 Inflammasome Activation

Damaged mitochondria release excessive reactive oxygen species, which serve as critical triggers for NLRP3 inflammasome assembly. We therefore examined whether muscone modulates mitochondrial reactive oxygen species (mt-ROS) as a functional readout of mitochondrial quality.

**Figure 4:**
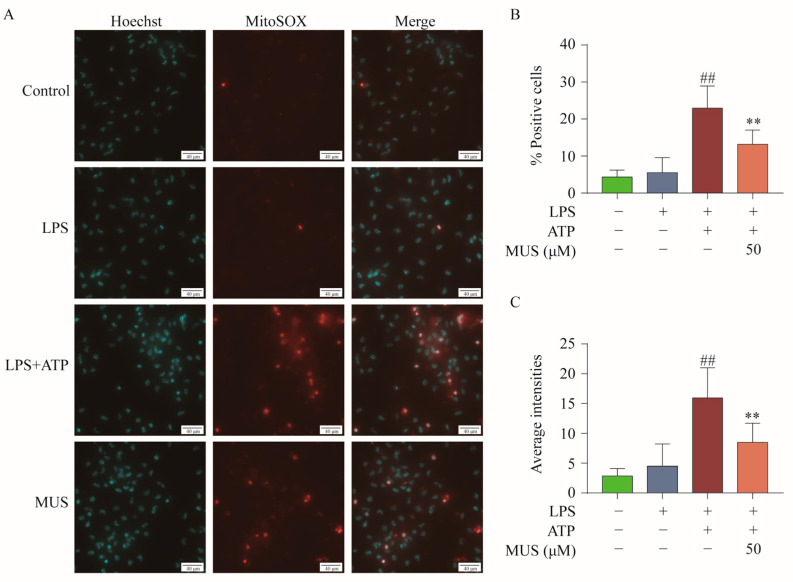
Muscone reduces mitochondrial ROS accumulation during NLRP3 inflammasome activation. LPS-primed PMs were treated with muscone (50 μM, 0.5 h prior to ATP stimulation). (**A**) MitoSOX Red staining of mt-ROS. Red: mt-ROS; blue: nuclei (Hoechst). Scale bar: 40 μm. (**B**) Percentage of mt-ROS-positive cells. (**C**) Mean fluorescence intensity. Representative images from three independent experiments are shown. Data are presented as mean ± SEM. ## *p* ≤ 0.01 vs. Control; ** *p* ≤ 0.01 vs. LPS + ATP using one-way ANOVA followed by Tukey’s multiple-comparison post hoc test.

MitoSOX Red staining revealed minimal mt-ROS elevation with LPS priming alone but robust accumulation following full NLRP3 activation (LPS + ATP)—in both the percentage of mt-ROS-positive cells and the mean fluorescence intensity. Muscone treatment during the activation stage (50 μM, 0.5 h prior to ATP) significantly dampened this mt-ROS surge (halving both the percentage of positive cells and mean fluorescence intensity; Figure 4A–C). This suppression is consistent with enhanced mitochondrial quality control, although not exclusive to mitophagy activation.

### 2.4. Muscone Promotes PINK1/Parkin-Associated Mitophagy During NLRP3 Inflammasome Activation

**Figure 5:**
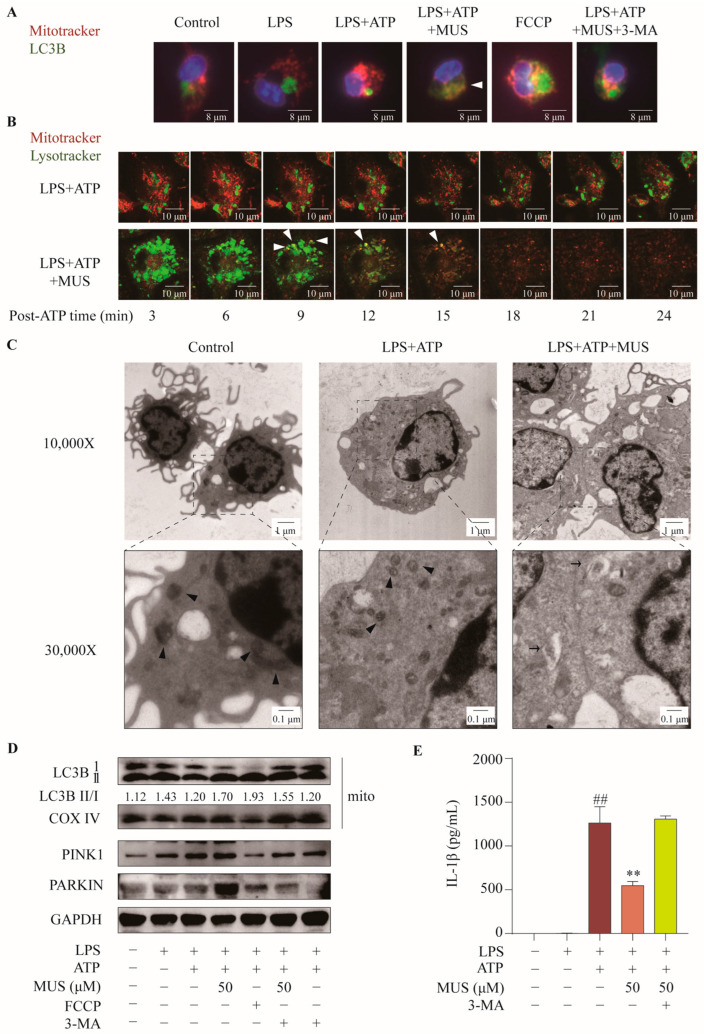
Muscone enhances PINK1/Parkin-associated mitophagy during NLRP3 inflammasome activation. LPS-primed PMs were pre-treated with muscone (50 μM, 0.5 h prior to ATP stimulation). FCCP (100 μM, 6 h) served as the positive control; 3-methyladenine (3-MA, 10 mM, 6 h), a PI3K inhibitor, served as the autophagy inhibitor. (**A**) Mitochondria–LC3B colocalization by immunofluorescence. Red: mitochondria (MitoTracker); green: LC3B; blue: nuclei (Hoechst). Scale bar: 8 μm. (**B**) Time-lapse live-cell imaging of mitochondria–lysosome fusion. Cells co-stained with MitoTracker Deep Red FM and LysoTracker Green. Time points indicate minutes post-ATP. Red: mitochondria; green: lysosomes. Scale bar: 10 μm. (**C**) Transmission electron microscopy. Upper panels: 10,000× (scale bar: 1 μm); lower panels: 30,000× (scale bar: 0.1 μm). Black arrowheads: cytoplasmic mitochondria; black arrows: mitochondria within autophagosomes. (**D**) Western blotting of mitochondrial fractions (LC3B-II, COX IV as loading control) and whole-cell lysates (PINK1, Parkin, GAPDH). (**E**) IL-1β levels in cell supernatants by ELISA. Representative images and blots from three independent experiments are shown. Data are presented as mean ± SEM. ## *p* ≤ 0.01 vs. Control; ** *p* ≤ 0.01 vs. LPS + ATP using one-way ANOVA followed by Tukey’s multiple-comparison post hoc test.

While mt-ROS suppression indicates improved mitochondrial quality, this finding alone is insufficient to establish mitophagy as the operative mechanism. We therefore applied multiple complementary approaches to directly assess mitophagic flux and its functional contribution to NLRP3 suppression. First, confocal microscopy revealed that muscone markedly increased mitochondria-LC3B colocalization compared to LPS + ATP-stimulated cells, an effect comparable to the mitophagy inducer FCCP and attenuated by the 3-methyladenine (3-MA, 10 mM), a phosphatidylinositol 3-kinase inhibitor that blocks autophagosome formation (Figure 5A). Time-lapse live-cell imaging further showed that muscone accelerated mitochondria–lysosome fusion, indicating enhanced mitophagic flux (Figure 5B). Transmission electron microscopy provided ultrastructural evidence: while control cells displayed intact mitochondrial cristae, LPS + ATP induced mitochondrial swelling and cristae disruption. Strikingly, muscone induced double-membraned autophagosomes engulfing damaged mitochondria—a morphological signature of canonical mitophagy (Figure 5C). At the molecular level, mitochondrial fractionation and Western blotting showed that muscone significantly increased mitochondrial LC3B-II recruitment, concomitant with upregulated PINK1 and Parkin expression in whole-cell lysates. Both effects were partially attenuated by 3-MA (Figure 5D). Finally, functional relevance was established by demonstrating that 3-MA co-treatment significantly reversed muscone’s suppression of IL-1β secretion, confirming that PINK1/Parkin-associated mitophagy contributes to muscone’s inhibition of NLRP3 inflammasome activation (Figure 5E). Collectively, these findings demonstrate that muscone activates PINK1/Parkin-associated mitophagy in macrophages, thereby removing damaged mitochondria and attenuating mt-ROS signals necessary for NLRP3 activation.

### 2.5. Muscone Enhances PINK1/Parkin-Associated Mitophagy in LPS-Challenged Mice

**Figure 6:**
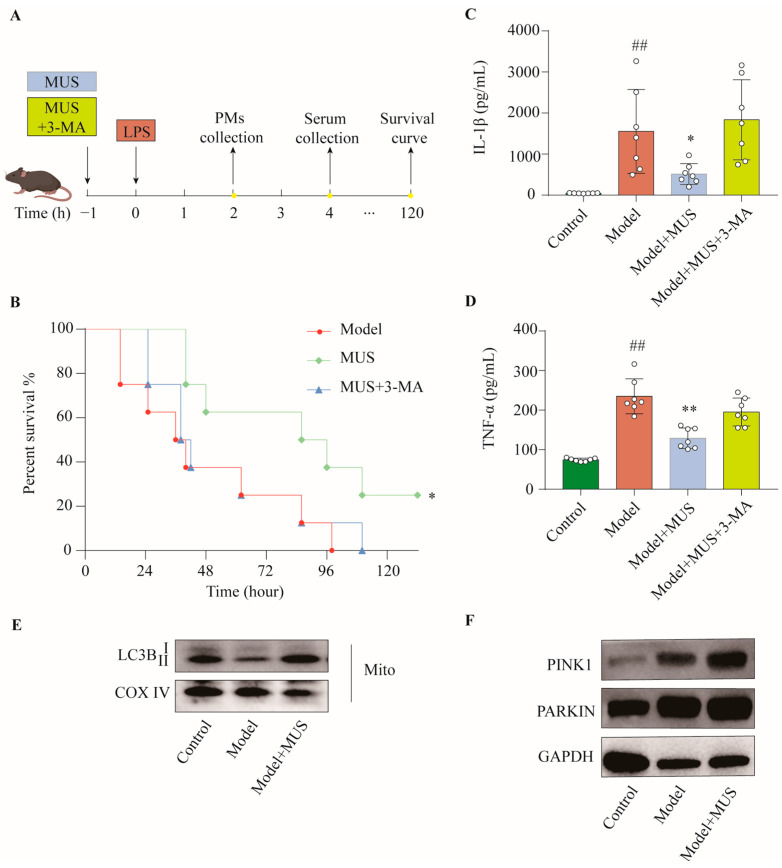
Muscone enhances PINK1/Parkin-associated mitophagy in LPS-challenged mice. (**A**) Experimental design: C57BL/6J mice received intraperitoneal (i.p.) LPS (25 mg/kg for survival, 20 mg/kg for cytokine and biochemical analyses) to induce endotoxemia. Muscone (30 mg/kg, i.p.) with or without 3-MA (15 mg/kg, i.p.) was administered 1 h before LPS challenge. (**B**) Survival curves (*n* = 8 per group), analyzed by log-rank test. (**C**,**D**) Serum IL-1β (**C**) and TNF-α (**D**) levels at 4 h post-LPS (*n* = 7). (**E**,**F**) Western blotting of peritoneal macrophages isolated at 2 h post-LPS (*n* = 3); (**E**) mitochondrial LC3B-II and COX IV (loading control). (**F**) PINK1, Parkin, and GAPDH (loading control). Representative blots from three independent experiments are shown. Data are presented as mean ± SEM. ## *p* ≤ 0.01 vs. Control; * *p* ≤ 0.05, ** *p* ≤ 0.01 vs. LPS using one-way ANOVA followed by Dunnett’s multiple-comparison post hoc test.

Finally, we tested in vitro findings in vivo, assessing whether muscone activates PINK1/Parkin-associated mitophagy in primary macrophages and protects against LPS-induced endotoxemia (Figure 6A). We found that muscone treatment significantly improved survival (3/8 mice alive at 98 h when all LPS controls had died; 2/8 survived to the 132 h endpoint), and this protective effect was partially reversed by co-administration of 3-MA (0/8 alive by 110 h) (Figure 6B). Concordantly, muscone markedly reduced serum levels of IL-1β and TNF-α (by ~70% and ~45%, respectively), effects largely reversed by 3-MA co-treatment (returning to near-baseline levels) (Figure 6C,D). Notably, analysis of peritoneal macrophages isolated from these mice revealed that muscone significantly enhanced mitochondrial recruitment of LC3B-II (Figure 6E) and upregulated the expression of PINK1 and Parkin (Figure 6F). Collectively, these findings demonstrate that muscone activates PINK1/Parkin-associated mitophagy in primary macrophages during endotoxemia, thereby linking enhanced mitochondrial quality control to improved survival and reduced inflammatory cytokine production.

## 3. Discussion

**Figure 7:**
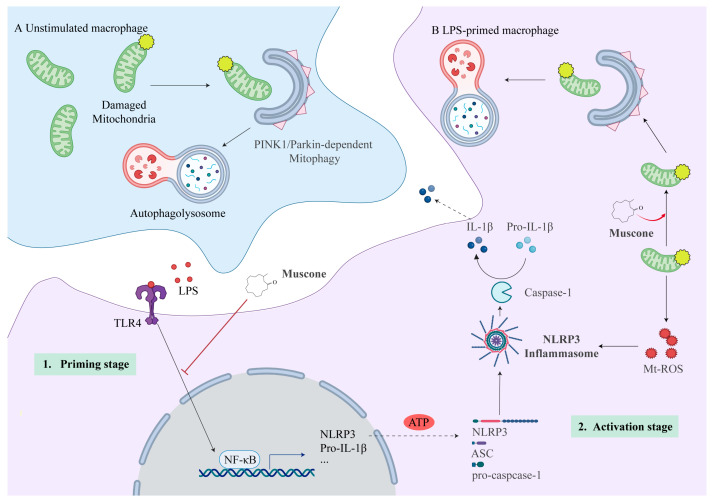
Schematic diagram illustrating the dual mechanism of muscone in suppressing NLRP3 inflammasome activation. (**A**) Under unstimulated conditions, PINK1/Parkin-associated mitophagy maintains mitochondrial homeostasis by clearing damaged mitochondria, ensuring mitochondrial quality control. (**B**) In LPS-primed macrophages, NF-κB activation upregulates NLRP3 and pro-IL-1β expression (priming). Concurrently, impaired PINK1/Parkin-associated mitophagy results in accumulation of damaged mitochondria and excessive mt-ROS, which fuels NLRP3–ASC–pro-caspase-1 complex assembly (activation). Muscone targets both processes: it inhibits NF-κB signaling to suppress priming and enhances PINK1/Parkin-associated mitophagy to eliminate damaged mitochondria and disrupt inflammasome assembly. (Created in BioRender. Qian, J. (2026) https://BioRender.com/nr89osk accessed on 15 May 2026).

In this study, we demonstrate that muscone inhibits NLRP3 inflammasome activation in macrophages and protects against LPS-induced endotoxemia by activating PINK1/Parkin-associated mitophagy—a mechanism previously unrecognized for this natural macrocyclic ketone. While muscone suppresses both NF-κB-dependent priming and inflammasome activation, its most distinctive feature lies in PINK1/Parkin-associated mitochondrial quality control, which eliminates the damage signal mt-ROS that fuels NLRP3 activation (Figure 7). These PINK1/Parkin-associated pro-mitophagy effects were identified by unbiased RNA sequencing and validated through fluorescence colocalization, transmission electron microscopy, and functional rescue with the autophagy inhibitor 3-MA. Furthermore, muscone significantly improved survival in vivo, with concomitant enhancement of PINK1/Parkin pathway markers in peritoneal macrophages. These findings establish muscone as a mitophagy-targeting NLRP3 inhibitor and highlight its macrocyclic scaffold as a promising lead for therapies directed at mitochondrial quality control in inflammatory diseases.

Building upon the growing body of muscone research, prior studies have established diverse mechanisms for muscone’s anti-inflammatory activity. For instance, Zhou et al. demonstrated that muscone attenuates neuroinflammation by regulating Drp1-dependent mitochondrial fission, a structural dynamics mechanism [16]. Separately, Yu et al. showed that muscone relieves inflammatory pain through inhibition of the NOX4/JAK2-STAT3 signaling cascade and subsequent NLRP3 inflammasome suppression in microglia [17]. Despite muscone’s broad pharmacological potential, as summarized in a recent comprehensive review [20], direct engagement of the mitochondrial quality control machinery—specifically PINK1/Parkin-associated mitophagy—in muscone-mediated NLRP3 inhibition has not been previously investigated. Our results establish this clearance-based mechanism as a previously unrecognized dimension of muscone pharmacology.

The development of NLRP3 inflammasome inhibitors has emerged as a priority for inflammatory diseases. Current candidates primarily target the protein complex directly: MCC950 directly binds the NLRP3 NACHT domain [21,22]; OLT1177 inhibits the NLRP3 ATPase activity [23]; BOT-4-one inhibits ATPase activity and promotes NLRP3 ubiquitination through alkylation [24]. To our knowledge, muscone represents the first natural macrocyclic ketone shown to target NLRP3 through organelle-level quality control. It achieves this by activating PINK1/Parkin-associated mitophagy, thereby eliminating proximal triggers such as damaged mitochondria and mt-ROS. This pharmacological enhancement of mitochondrial quality control recapitulates the physiological protective role of the PINK1/Parkin pathway, supported by observations that genetic deficiency of this axis exacerbates inflammation across disease models [11,25]. Unlike strategies that prevent mitochondrial damage [26], muscone acts through active elimination of damaged organelles, representing a distinct clearance-based mitochondrial quality control mechanism for NLRP3 suppression.

While our findings establish PINK1/Parkin-associated mitophagy as the operative mechanism for muscone’s anti-inflammatory effects, its precise molecular target remains to be identified. Candidate targets include PINK1, Parkin, or upstream regulators such as TSPO (implicated by our RNA-seq data). Resolving this will require direct binding assays, photoaffinity labeling coupled with mass spectrometry, or cryo-EM structural analysis. Furthermore, genetic validation of PINK1/Parkin dependency (e.g., siRNA or CRISPR-mediated knockdown) was not performed in this study and remains an important priority for future mechanistic investigation. Additionally, although muscone significantly improved survival and attenuated inflammatory cytokine production in the acute endotoxemia mouse model, translation to human patients remains premature and requires extensive preclinical and clinical development.

The macrocyclic scaffold of muscone provides an attractive yet underexplored foundation for structure–activity relationship studies. Three strategic priorities address distinct limitations of the native compound: (i) introducing polar substituents to improve aqueous solubility—critical for parenteral and oral formulations—while preserving the lipophilicity required for blood–brain barrier permeability and central nervous system access; (ii) rigidifying the flexible fifteen-membered ring to reduce volatility and enhance chemical stability under storage and manufacturing conditions, with the added potential of locking bioactive conformations for improved target engagement; and (iii) exploring modifications at the C-3 methyl position to optimize pharmacophore interactions with the PINK1/Parkin axis, thereby maximizing mitophagy activation and NLRP3 suppression. Such optimized analogs may extend therapeutic utility beyond acute endotoxemia to chronic conditions characterized by PINK1/Parkin dysfunction, including Parkinson’s disease, cardiac ischemia–reperfusion injury, and age-related inflammatory disorders. A complementary strategy would combine mitophagy-targeting agents with direct NLRP3 inhibitors.

## 4. Materials and Methods

### 4.1. Chemicals and Reagents

Muscone (CAS: 541-91-3, B21154), ATP (B25057), carbonyl cyanide 4-(trifluoromethoxy) phenylhydrazone (FCCP, S80686), and 3-methyladenine (3-MA, B25357) were purchased from Yuanye Biotechnology (Shanghai, China). Lipopolysaccharide (LPS, L2630) was purchased from Sigma-Aldrich (St. Louis, MO, USA). Protein A/G magnetic beads (HY-K0202) were obtained from MedChemExpress (Monmouth Junction, NJ, USA). Fluorescent probes used in this study included MitoSOX Red mitochondrial superoxide indicator (Yeasen, Shanghai, China, 720827), MitoTracker^®^ Deep Red FM (Yeasen, 40743), Lyso-Tracker Green (Beyotime, Shanghai, China, C1047S), and Hoechst 33342 (Cell Signaling Technology, Danvers, MA, USA, 4082). Primary antibodies against pro-caspase-1 (sc-56036), PINK1 (sc-33796), and Parkin (sc-30130) were purchased from Santa Cruz Biotechnology (Dallas, TX, USA). Antibodies against NLRP3 (15101S), ASC (67824S), NF-κB p65 (8242S), p-NF-κB p65 (3033S), and LC3B (83506S) were purchased from Cell Signaling Technology. Anti-pro-IL-1β antibody (AF-401-NA) was purchased from R&D Systems (Minneapolis, MN, USA). Anti-COX IV (AF6549), anti-tubulin (AF5012), and anti-GAPDH (AF5009) antibodies were obtained from Beyotime Biotechnology.

### 4.2. Animal Studies

C57BL/6J mice (6–8 weeks old) were obtained from Zhejiang Vital River Laboratory Animal Technology Co., Ltd. (Jiaxing, China) or SLAC Laboratory Animal Co., Ltd. (Shanghai, China) for in vivo studies and macrophage isolation, respectively. Mice were housed under a 12 h light/dark cycle with ad libitum access to food and water in a 20 ± 2 °C environment. All animal experiments were performed using male C57BL/6J mice to minimize variability associated with estrous cycle fluctuations in female mice, which can influence inflammatory responses and cytokine profiles.

Mice were allocated to experimental groups by simple randomization. Three independent in vivo experiments were conducted with a four-group design: Control, LPS, LPS + muscone, and LPS + muscone + 3-MA. Experiment 1 assessed survival (*n* = 8 per group); Experiment 2 measured serum cytokines at 4 h post-LPS (*n* = 7 per group); and Experiment 3 evaluated mitophagy protein expression in peritoneal macrophages at 2 h post-LPS (*n* = 3 per group).

The LPS-induced endotoxemia mouse model was established as previously described [18]. In brief, mice received an intraperitoneal (i.p.) injection of LPS (25 mg/kg for survival analysis; 20 mg/kg for other endpoints), while mice in the Control group received an equivalent volume of sterile saline. Muscone (30 mg/kg i.p.; dissolved in sterile saline to form a stock solution at 3 mg/mL) was administered 1 h prior to LPS challenge, with the dose determined based on previous reports [27]. To verify the involvement of autophagy, the inhibitor 3-MA (15 mg/kg, i.p.; dissolved in a muscone stock solution to form a stock solution at 1.5 mg/mL) [28] was co-administered with muscone.

For survival analysis, mice were monitored every 12 h, with more frequent observations when clinical deterioration was apparent, until the 132 h endpoint. For serum cytokine analysis, blood was collected at 4 h post-LPS challenge. For mitophagy protein analysis, peritoneal macrophages were harvested at 2 h post-LPS [29]. Experiments were performed by one investigator blinded to group allocation; data were analyzed by a second independent investigator. After completion of each experiment, all mice were euthanized.

### 4.3. Cell Preparation and Culture

Peritoneal macrophages (PMs) were isolated as previously described [30]. Briefly, mice were administered an i.p. injection of 1 mL of 3% (*w*/*v*) sterile thioglycolate medium (Sigma-Aldrich). Five days post-injection, cells were harvested by peritoneal lavage, filtered through a 70 μm cell strainer, and red blood cells were removed using an ammonium chloride solution (Stemcell Technologies, Vancouver, BC, Canada, 07800). The remaining PMs were then seeded into culture plates and incubated overnight in RPMI 1640 medium supplemented with 10% heat-inactivated fetal bovine serum (FBS) and 1% penicillin/streptomycin. All cultures were maintained at 37 °C in a humidified atmosphere containing 5% CO_2_ prior to experimental intervention.

### 4.4. NLRP3 Inflammasome Activation and Drug Treatment

The LPS/ATP-stimulated PM model was employed to examine NLRP3 inflammasome activation as previously described [26]. In brief, PMs were primed with LPS (500 ng/mL) for 5.5 h, followed by activation with ATP (2 mM) for another 0.5 h. Muscone (12.5, 25, or 50 μM, diluted in RPMI 1640 medium) was administered either 0.5 h before LPS (designated as “Before”; mimic priming stage) or 5 h after LPS challenge (designated as “After”; mimic activation stage). For mechanistic validation, FCCP (100 μM) was used as a positive mitophagy inducer [31], while 3-MA (10 mM) served as an autophagy inhibitor [32]. At indicated time points, culture supernatants were collected for cytokine analysis, and cells were harvested for RNA-seq and subsequent functional assays.

### 4.5. Enzyme-Linked Immunosorbent Assay (ELISA)

Mouse serum and cell culture supernatants were collected and centrifuged at 3000 rpm for 10 min at 4 °C to remove cellular debris. The concentrations of IL-1β and TNF-α were measured using Mouse Uncoated ELISA Kits (Thermo Fisher Scientific, Waltham, MA, USA; IL-1β, 88-7013-88; TNF-α, 88-7324-88) according to the manufacturer’s instructions.

### 4.6. Western Blotting

PMs were lysed in IP lysis buffer (Beyotime, P0037) on ice. Lysates were centrifuged at 12,000 rpm for 10 min at 4 °C, and the supernatants were collected and used as total cell lysates. Mitochondria were isolated using a Cell Mitochondria Isolation Kit (Beyotime, C3601) according to the manufacturer’s instructions. Lysates were mixed with 4× Sample Buffer (Bio-Rad, Hercules, CA, USA, 161-0747) and boiled for 5 min. Proteins were separated by sodium dodecyl sulfate–polyacrylamide gel electrophoresis (SDS-PAGE) and transferred to polyvinylidene difluoride (PVDF) membranes (Millipore, Billerica, MA, USA, IPVH00010). Membranes were blocked with 5% (*w*/*v*) non-fat milk (BBI Life Sciences, Shanghai, China, A600669) in 1 × TBST, incubated with primary antibodies (1:1000) overnight at 4 °C, followed by incubation with secondary antibodies (1:10,000) at room temperature for 1 h. Protein bands were detected using an enhanced chemiluminescence (ECL) system and visualized on a ChemiDoc Touch Imaging System (Bio-Rad).

### 4.7. ASC Oligomerization and ASC Speck Formation

ASC oligomerization and ASC speck formation assays were carried out as previously described [18]. In brief, for oligomerization detection, cell lysates were cross-linked with 2 mM suberic acid bis(N-hydroxysuccinimide ester) (Sigma-Aldrich, S1885) for 30 min and then centrifuged at 5000× *g* for 10 min at 4 °C. Pellets were mixed with 4× Sample Buffer (Bio-Rad) and analyzed by Western blotting; non-crosslinked lysates served as input controls.

For ASC speck analysis, PMs were fixed with 4% paraformaldehyde (BBI, E672002), permeabilized with 0.1% Triton X-100 (Sigma-Aldrich, T8787), and blocked with 5% bovine serum albumin (BSA, BBI, A600332) for 1 h at room temperature. Cells were incubated with an anti-ASC antibody (1:500) overnight at 4 °C, followed by incubation with a FITC-conjugated secondary antibody (1:200) for 1 h at room temperature. Nuclei were counterstained with Hoechst 33342 (CST, 2 μg/mL) for 10 min before imaging. Images were captured using an ImageXpress Pico (Molecular Devices, San Jose, CA, USA) with a 40× objective. ASC speck-positive cells were quantified as follows: (ASC speck-positive cells)/(total cells) × 100.

### 4.8. Co-Immunoprecipitation (Co-IP)

Co-IP was performed to assess the interaction between pro-caspase-1 and ASC. Cell lysates were incubated with anti-ASC antibodies and protein A/G magnetic beads (MedChemExpress) following the manufacturer’s instructions. After washing, bound proteins were eluted in 4× Sample Buffer (Bio-Rad) by boiling for 10 min. Pro-caspase-1 and ASC levels were analyzed by Western blotting; whole-cell lysates served as input controls.

### 4.9. RNA Sequencing (RNA-Seq)

Cells from the Control, LPS + ATP, and muscone (50 μM, activation stage) groups were collected and sent to Novogene (Beijing, China) for RNA extraction and sequencing (*n* = 2 per group). Raw sequencing data were deposited in the Gene Expression Omnibus (GEO) database (https://www.ncbi.nlm.nih.gov/gds; accessed on 10 December 2025; accession number: GSE313142). Gene expression was quantified as fragments per kilobase of transcript per million mapped reads (FPKM) using RSEM. Differentially expressed genes (DEGs) were identified using DESeq2 (v1.20.0) with the following criteria: $\left|log2(foldchange)\right|>1$ and adjusted *p*-value ≤ 0.05. Shared and unique DEGs among groups were visualized using bar plots and Venn diagrams. Hierarchical clustering was performed to display the expression patterns of shared DEGs. Functional enrichment analyses of shared DEGs were conducted using the Kyoto Encyclopedia of Genes and Genomes (KEGG) and Gene Ontology (GO) databases. Detailed RNA-seq analysis parameters are provided in Table S1.

### 4.10. Detection of mt-ROS

Mitochondrial ROS production was assessed using a MitoSOX Red mitochondrial superoxide indicator (Yeasen). Briefly, after stimulation, cells were incubated with 3 μM MitoSOX in RPMI 1640 medium for 10 min at 37°C, followed by nuclear counterstaining with Hoechst 33342 (2 μg/mL) for 10 min. Fluorescence images were acquired using an ImageXpress Pico automated cell imaging analysis system (Molecular Devices, San Jose, CA, USA) with a 40× objective. The percentage of mt-ROS-positive cells and the average intensity per group were quantified using the ImageXpress Pico automated cell imaging analysis system.

### 4.11. Fluorescence Colocalization Analyses of Mitochondria with LC3B and Lysosomes

For the detection of mitochondria-LC3B colocalization, cells were incubated with 0.25 μM MitoTracker Deep Red FM for 20 min at 37 °C, then fixed with ice-cold methanol (Merck, Darmstadt, Germany, 1060095000) for 5 min at −20 °C. After blocking with 5% BSA for 1 h at room temperature, cells were incubated with an anti-LC3B primary antibody (1:500) overnight at 4 °C, followed by incubation with a FITC-conjugated secondary antibody (1:200) for 1 h at room temperature. Nuclei were counterstained with Hoechst 33342 (2 μg/mL) for 10 min. Images were acquired using an ImageXpress Pico automated imaging system with a 63× objective and analyzed using the ImageXpress PICO automated cell imaging analysis system.

To monitor the colocalization of mitochondria and lysosomes, LPS-primed cells were co-stained with 0.25 μM MitoTracker Deep Red FM for 20 min, followed by incubation with 50 nM LysoTracker Green for 15 min at 37 °C. After washing, cells were stimulated with ATP (2 mM) and imaged every 3 min using a Leica TCS SP8 laser scanning confocal microscope (100× objective) equipped with 488 and 640 nm excitation lasers (Leica Microsystems, Wetzlar, Germany).

### 4.12. Transmission Electron Microscopy

For ultrastructural analysis of mitophagy, cells were fixed in 2.5% glutaraldehyde (Sinopharm, Shanghai, China, 30092436) overnight at 4 °C, embedded in 2% agarose (Sinopharm, 68000133), post-fixed with 1% osmium tetroxide (SPI Supplies, West Chester, PA, Z02601) for 1 h at room temperature, and dehydrated through a graded ethanol series (Sinopharm, FA9954F4L). Samples were then embedded in Spurr resin (SPI Supplies, 02680-AB), sectioned into 70 nm slices, and stained with uranyl acetate (SPI Supplies, 02624-AB) and lead citrate (Sinopharm, 200964701). Ultrastructure was examined using a Hitachi H-7650 transmission electron microscope (Hitachi, Tokyo, Japan).

### 4.13. Statistical Analyses

Statistical analyses were performed using GraphPad Prism 10.1.2 (San Diego, CA, USA). Normality (Shapiro–Wilk) and homoscedasticity (Brown–Forsythe) were verified. Parametric data were compared using one-way analysis of variance (ANOVA) followed by Tukey’s multiple-comparison post hoc test among all groups and Dunnett’s multiple-comparison post hoc test against a single group. Survival rates were analyzed by the Kaplan–Meier method and compared using the log-rank test. Data are expressed as the mean ± standard error of the mean (SEM). Statistical significance was set at *p* ≤ 0.05.

## 5. Conclusions

In summary, we identify muscone as a pharmacological enhancer of PINK1/Parkin-associated mitophagy that suppresses NLRP3 inflammasome activation in macrophages and improves survival in an acute endotoxemia model. These findings establish proof-of-concept for organelle-level quality control as an anti-inflammatory strategy. Future priorities include (i) genetic validation of PINK1/Parkin dependency using siRNA or CRISPR approaches; (ii) identification of precise molecular targets through direct binding assays and structural analysis; (iii) optimization of pharmacokinetic properties and evaluation of long-term safety; and (iv) assessment of therapeutic potential in chronic disease models.

## Data Availability

The original contributions presented in this study are included in the article and Supplementary Material. Further inquiries can be directed to the corresponding authors.

## Supplementary Materials

The following supporting information can be downloaded at: https://www.mdpi.com/article/10.3390/ph19060816/s1, Figure S1: Muscone-MTT; Table S1: RNA-seq data.

## Abbreviations

The following abbreviations are used in this manuscript:

NLRP3	NOD-like receptor protein 3
ASC	apoptosis-associated speck-like protein containing a CARD
LPS	lipopolysaccharide
NF-κB	nuclear factor kappa B
IL-1β	interleukin-1β
TNF-α	tumor necrosis factor-α
3-MA	3-methyladenine
FCCP	carbonyl cyanide 4-(trifluoromethoxy)phenylhydrazone
LC3B-II	microtubule-associated protein 1 light chain 3B-II
mt-ROS	mitochondrial reactive oxygen species
PINK1	PTEN-induced putative kinase 1
