# Supplementary material for: Muscone Promotes PINK1/Parkin-Associated Mitophagy to Suppress NLRP3 Inflammasome Activation: Implications for Endotoxemia Therapy
Source: Pharmaceuticals (Basel). 2026 May 23;19(6):816. doi: 10.3390/ph19060816 (PMC13305954; doi:10.3390/ph19060816)
Supplement: Supplementary file 1 [file pharmaceuticals-19-00816-s001.zip › Figure S1.pdf]

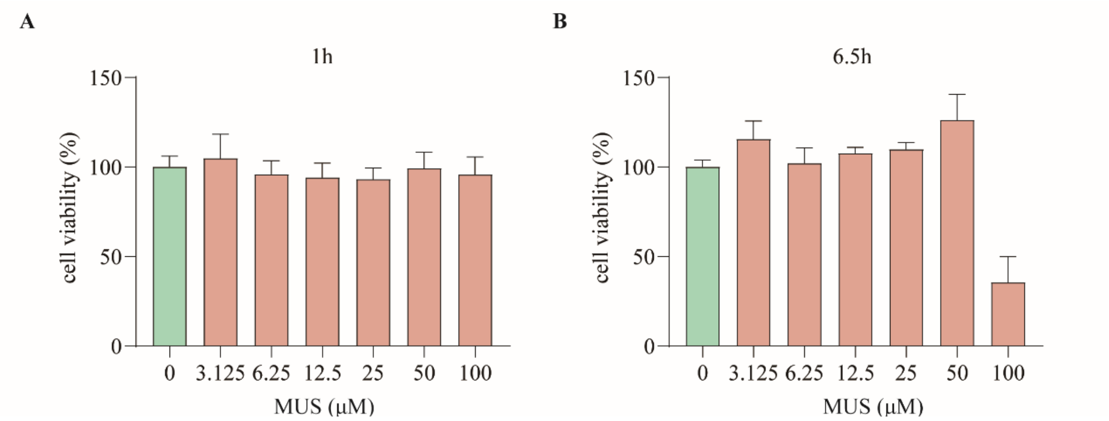

Figure S1. The effects of muscone on the viability of peritoneal macrophages. Peritoneal macrophages were treated with different concentrations of muscone for 1 h or 6.5 h, respectively. Cell viability was assessed by MTT. (**A**) 1 h; (**B**) 6.5 h. Data are presented as mean ± standard error of the mean (SEM) from three independent experiments.
